# Supplementary material for: Mesenchymal Stromal Cell‐Mediated Intercellular Communication: Mapping the Interactome for Skeletal Muscle Homeostasis and Regeneration
Source: Adv Sci (Weinh). 2026 May 29;13(46):e07444. doi: 10.1002/advs.202507444 (PMC13336089; doi:10.1002/advs.202507444)
Supplement: Supplementary file 1 [file ADVS-13-e07444-s001.docx]

**Supplementary figures**

**Supplementary Figure 1. Graphical Abstract.**

**Supplementary Figure 2. Identifying cellular populations in homeostatic and regenerating SkM.** (A, B) Heatmaps showing the top 10 enriched features in each population in (A) uninjured and (B) injured SkM resolved with the FindAllMarkers function. Populations in the heatmap are noted by corresponding color to the right panel. (C) Top 10 differentially altered biological processes in FAPs from uninjured SkM (left) and SkM at 3DP (right), comparing FAP-depleted (DP) versus control (CT) samples. (D, E) Gating strategy for identifying cellular fractions in SkM. D: identifying SCs and FAPs; E: Identifying ICs and ECs. (F) Gating strategy for identifying monocytes in bone marrow. SC - satellite cells, FAPs - fibroadipogenic progenitors, ICs - immune cells, ECs - endothelial cells, Teno - tenocytes, Neut - neutrophils, SMC - smooth muscle cells, Mo/Mφs - Monocytes/Macrophages, B - B cells, NK/T - NK cells/T cells, Sch - Schwann cells, APC - antigen-presenting cells, Unk - Unknown, PB - Pacific Blue, CT - control, DP - FAP-depleted.

**Supplementary Figure 3. Elucidating FAP-dependent gene expression and molecular pathways in cells of the homeostatic niche.** (A) UMAPs of FAPs after independently reclustering from Fig. 2A. Cells are colored by noted subpopulation identity (top) or sample (bottom). (B) Volcano plots representing DEGs in the noted cell populations from control and FAP-depleted samples. Red: DEGs with log_2_FC > 0.5 and adjusted p-value < 0.05. Blue: DEGs with log_2_FC ≤ 0.5 and adjusted p-value < 0.05. Gray: DEGs with adjusted p-value ≥ 0.05. (C-E) Top 10 upregulated and downregulated biological processes in noted cell populations in uninjured SkM, comparing FAP-depleted (DP) versus control (CT) samples.

**Supplementary Figure 4. FAP interactions in homeostatic SkM**. (A, B) Chord plots from CellChat analyses summarize the interactions between cells of homeostatic SkM. (A), and resolved by pathway types (B). The thickness of the lines represents the number of interactions (left) and interaction weights/strength (right).

**Supplementary Figure 5.** **Molecular influence of FAPs on subpopulations of SCs and Mo/Mφs in SkM homeostasis.** (A) UMAPs of SCs after independent reclustering of the parent population in Fig. 2A. Subpopulations are color-coded, corresponding with colors in the enrichment score plot (right) of SC subclusters in uninjured SkM. Positive scores represent a greater abundance of cells in FAP-depleted mice, while negative scores indicate a greater abundance in control mice. (B) Heatmap showing the top 10 enriched features in each (A) subpopulation in SCs in uninjured SkM resolved with the FindAllMarkers function. Specific markers are listed in Supplementary Table 1. (C) Top 10 biological processes in SC cluster 2, compared to other SC subclusters. (D) Dot plot representing the expression of genes associated with myogenesis across SC populations in uninjured SkM. (E) UMAPs of Mo/Mφs after independently reclustering their parent population from Fig. 2A. Subpopulations are color-coded, corresponding to their noted identity in the enrichment score plot of Mo/Mφ subclusters in uninjured SkM (right). Positive scores represent a greater abundance of cells in FAP-depleted mice, while negative scores indicate a greater abundance in control mice. (F) Heatmap showing the top 10 enriched features in each subpopulation of Mo/Mφs in uninjured SkM resolved with the FindAllMarkers function. Specific markers are listed in Supplementary Table 2. (G) Top 10 biological enriched processes in noted Mo/Mφ subpopulations versus other subpopulations.

**Supplementary Figure 6.** **Molecular impact of FAPs on neutrophil subpopulations in homeostasis.** (A) UMAPs of neutrophils after independently reclustering from their parent population in Fig. 2A. Cells are color-coded by subpopulation identity, with corresponding the enrichment score plot of neutrophils subclusters in uninjured SkM (right). Positive scores represent a greater abundance of cells in FAP-depleted mice, while negative scores indicate a greater abundance in control mice. (B) Dot plot representing the abundance and expression level of genes associated with neutrophil developmental stages across neutrophil populations in uninjured SkM. (C) UMAPs of neutrophils are created by Monocle 3, with cells colored by the neutrophil developmental stage. (D) UMAPs of neutrophils created by Monocle 3, with cells colored by pseudotime. Immature neutrophils were manually set as the root of the main trajectory. Most proliferating neutrophils were in a minor separated trajectory (gray). (E) Expression of marker genes indicating neutrophil developmental stage along pseudotime. (F) Abundance of neutrophil subpopulations along pseudotime. (G) Top 10 biological processes in noted neutrophil subpopulations, compared to others. (H) Interactions between FAPs (sender) and neutrophils (receiver). The thickness of lines represents interaction strength. (I) Left: Interactions between Mo/Mφs (sender) and neutrophils (receiver). The thickness of lines represents interaction strength. Right: Dot plot representing the abundance and expression level of genes associated with chemokines in Mo/Mφs across samples in uninjured SkM. (J) Left: Interactions between neutrophils (sender) and Mo/Mφs (receiver). The thickness of lines represents interaction strength. Right: Dot plot representing the abundance and expression level of genes associated with chemokines in neutrophils across samples in uninjured SkM.

**Supplementary Figure 7. FAP-dependent molecular pathways across cell populations in SkM regeneration.** (A-F) Top 10 upregulated and downregulated biological processes in noted cell populations in 3 dpi SkM, comparing FAP-depleted (DP) versus control (CT) samples. Truncated term in (A) “reactions with bulged adenosine as nucleophile” = RNA splicing, via transesterification reactions with bulged adenosine as nucleophile. DP - FAP-depleted, CT - control; SCs - satellite cells, Teno - tenocytes, EC - endothelial cells, Mo/Mφs - monocytes/macrophages, APC - antigen-presenting cells, Neut - neutrophils.

**Supplementary Figure 8.** **Regulatory potential of FAP-derived ligands for different cell populations in SkM regeneration.** (A) Top 10 FAP-derived ligands in 3 dpi SkM and their activity scores quantitated by the area under the precision-recall curve (AUPR), with noted cell populations set as receivers. (B) Dot plot representing the abundance and expression level of genes encoding receptors of FGF7 (Fgfr1, Fgfr4, Nrp1) and IL-6 (Il6ra, Il6st) across cell populations in SkM at 3 dpi.

**Supplementary Figure 9. FAP interactions in regenerating SkM**. (A, B) Chord plots from CellChat analyses summarize the interactome between cells of regenerating SkM (A), and resolved by pathway types (B). The thickness of the lines represents the number of interactions (left) and interaction weights/strength (right).

**Supplementary Figure 10.** **SC heterogeneity in SkM regeneration.** (A) UMAPs focused on the FAP, SC, and tenocyte populations in SkM regeneration (Fig. 4A) with the expression level of noted myogenic genes. Note, that FAP-depleted and control cells are overlaid in this representation. Purple gradient - feature enrichment score. (B) UMAP showing the distribution of cells in SC subclusters 0, 3, and 4 mapping to the parent UMAP from Fig. 4A. (C) UMAPs derived from scRNA-seq of cells from FAP-containing and depleted SkM at 3 dpi (left). UMAP of FAPs, SCs, and tenocytes after independently reclustering from the left panel, with cells colored by subcluster (middle). The dot plot (right) represents gene abundance and expression level for cluster identification across cell types in SkM at 3 dpi. (D) UMAPs of FAPs, SCs, and tenocytes after independently reclustering, with cells colored by their original identities in the parent UMAP (C, left).

**Supplementary Figure 11. FAP-dependent regulatory mechanisms of tenocyte and endothelial cell activity in SkM regeneration.** (A) Top FAP-derived ligands with the highest activity and their predicted impact on DEGs in tenocytes, with the dot plot showing the abundance and expression level of these ligands across cell types in SkM at 3 dpi. (B) Interactions between FAPs (sender) and tenocytes (receiver). The thickness of lines represents interaction strength. (C) Top 10 biological processes in tenocytes inferred to be regulated by Fgf7 (left) and Il6 (right). (D) Top FAP-derived ligands with the highest activity and their predicted impact on DEGs in ECs, with the dot plot showing the abundance and expression level of these ligands across cell types in SkM at 3 dpi. (E) Interactions between FAPs (sender) and ECs (receiver). The thickness of lines represents interaction strength. (F) Top 10 biological processes in ECs inferred to be regulated by Fgf7 (left) and Il6 (right). Truncated terms in (B) and (E) “ITGA5” = ITGA5_ITGB1, “ITGAV” = ITGAV_ITGB1, and “ITGA6” = ITGA6_ITBG1.

**Supplementary Figure 12. FAP-dependent regulatory mechanisms of neutrophil and mononuclear phagocyte activity in SkM regeneration** (A) Top FAP-derived ligands with the highest activity and their predicted impact on DEGs in neutrophils, with the dot plot showing the abundance and expression level of these ligands across cell types in SkM at 3 dpi. (B) Interactions between FAPs (sender) and neutrophils (receiver). The thickness of lines represents interaction strength. (C) Top 10 biological processes in neutrophils (Neut) inferred to be regulated by Il6. (D) Heatmap showing the top 10 enriched features in each subpopulation of mononuclear phagocytes in regenerating SkM (3 dpi) resolved with the FindAllMarkers function. Specific markers are listed in Supplementary Table 3. (E) Dot plot representing the abundance and expression level of genes for subcluster identification across mononuclear phagocytes in SkM at 3 dpi. (F) UMAPs of cells in SkM at 3 dpi overlaid with the expression level of Ccrl2 in the noted samples. Inserts indicate the identities of mononuclear phagocytes by colors. Red - inflammatory Mo/Mφs; yellow - Gpnmb+ macrophages; green - regenerative macrophages; blue - APCs. Background UMAP is from Fig. 4A. (G, H) Interactions between FAPs (sender) and (G) Gpnmb+ macrophages and (H) regenerative macrophages (receivers). The thickness of lines represents interaction strength. The truncated term “TREM2_” = TREM2_TYROBP. (I) Dot plot representing the abundance and expression level of genes associated with proliferation in regenerative macrophages across cell types in SkM at 3 dpi. (J) Violin plots represent gene expression levels associated with proliferation in regenerative macrophages, split by control or FAP-depleted (Depleted) sample.

**Supplementary Figure 13. SC responses to FAP-CM and IL-6 neutralization.** (A) Effect of IL-6 neutralization on SC proliferation cultured in BM or CM with indicated concentrations of αIL-6. n = 6 per condition. BM (0), and CM (0, 0.1, 1.0, 10) data are from Fig. 8C. (B) Distribution of nuclear Pax7 and MyoD fluorescence intensities in SCs cultured in BM or CM with noted αIL-6 treatment. Violin plots represent single-cell measurements pooled across replicates. n = 883-1199 cells pooled over 3 replicates. (C) Quantification of SC state based on Pax7 and MyoD expression in BM or CM treated cultures with indicated concentrations of αIL-6. Pax7+MyoD- (quiescent-like), Pax7+MyoD+ (activated/proliferating), and Pax7-MyoD+ (differentiated; rare and not readily visualized in graph). n = 3 per condition. Significance comparisons were made relative to BM counterparts. BM (0), and CM (0, 0.1, 1.0, 10) data are from Fig. 8F. Error bars represent mean ± SEM; statistical tests were performed using unpaired Student’s t-tests. *p < 0.05, ** p < 0.01, **** p < 0.0001, ns = not significant.

**Supplementary Figure 14. Monocyte survival and state in response to FAP-CM and IL-6 neutralization.** (A) Representative brightfield images of primary monocytes cultured in BM or CM for 12 or 72 hr demonstrating the loss of cells in BM. Representative flow cytometry plots indicate gating strategy for discriminating living cells from debris. Scale bar, 20 μm. (B) Quantification of live monocytes per well cultured in BM or CM with increasing concentrations of IL-6 neutralizing antibody. n = 3 per condition. BM (0), and CM (0, 0.1, 1.0, 10) data are from Fig. 9C. (C) Representative flow cytometry plots showing the gating strategy for identifying monocyte populations across stages of progression toward macrophage differentiation. Normalized to Volume = absolute cell number/31.5 µL for each condition; Normalized to Events = relative proportion/150 cells for each condition. Error bars represent mean ± SEM; statistical tests were performed using unpaired Student’s t-tests. *p < 0.05, ** p < 0.01.

**Supplementary Figure 15. A FAP-centered interactome in homeostatic and regenerative SkM.** Predicted ligand-receptor interactions inferred from the FAP interactome analysis, providing a framework to prioritize candidate signaling relationships for further functional investigation.

**References**

1. Frontera WR, Ochala J. Skeletal Muscle: A Brief Review of Structure and Function. *Calcif Tissue Int*. 2015;96(3):183-195. doi:10.1007/s00223-014-9915-y

2. Shearer J, Graham TE. New Perspectives on the Storage and Organization of Muscle Glycogen. *Can J Appl Physiol*. 2002;27(2):179-203. doi:10.1139/h02-012

3. Davis T. Contribution of skeletal muscle to nonshivering thermogenesis in the dog. *Am J Physiol-Leg Content*. 1967;213(6):1423-1426. doi:10.1152/ajplegacy.1967.213.6.1423

4. Collins CA, Olsen I, Zammit PS, et al. Stem Cell Function, Self-Renewal, and Behavioral Heterogeneity of Cells from the Adult Muscle Satellite Cell Niche. *Cell*. 2005;122(2):289-301. doi:10.1016/j.cell.2005.05.010

5. Menshikova EV, Ritov VB, Ferrell RE, Azuma K, Goodpaster BH, Kelley DE. Characteristics of skeletal muscle mitochondrial biogenesis induced by moderate-intensity exercise and weight loss in obesity. *J Appl Physiol*. 2007;103(1):21-27. doi:10.1152/japplphysiol.01228.2006

6. Brooks SV, Faulkner JA. Contractile properties of skeletal muscles from young, adult and aged mice. *J Physiol*. 1988;404(1):71-82. doi:10.1113/jphysiol.1988.sp017279

7. Brooks SV, Faulkner JA. Contraction-induced injury: recovery of skeletal muscles in young and old mice. *Am J Physiol-Cell Physiol*. 1990;258(3):C436-C442. doi:10.1152/ajpcell.1990.258.3.c436

8. Fielding RA, Vellas B, Evans WJ, et al. Sarcopenia: An Undiagnosed Condition in Older Adults. Current Consensus Definition: Prevalence, Etiology, and Consequences. International Working Group on Sarcopenia. *J Am Méd Dir Assoc*. 2011;12(4):249-256. doi:10.1016/j.jamda.2011.01.003

9. Irintchev A, Zweyer M, Wernig A. Impaired functional and structural recovery after muscle injury in dystrophic mdx mice. *Neuromuscul Disord*. 1997;7(2):117-125. doi:10.1016/s0960-8966(96)00422-1

10. Yin H, Price F, Rudnicki MA. Satellite Cells and the Muscle Stem Cell Niche. *Physiol Rev*. 2013;93(1):23-67. doi:10.1152/physrev.00043.2011

11. Joe AWB, Yi L, Natarajan A, et al. Muscle injury activates resident fibro/adipogenic progenitors that facilitate myogenesis. *Nat Cell Biol*. 2010;12(2):153-163. doi:10.1038/ncb2015

12. Wosczyna MN, Konishi CT, Carbajal EEP, et al. Mesenchymal Stromal Cells Are Required for Regeneration and Homeostatic Maintenance of Skeletal Muscle. *Cell Rep*. 2019;27(7):2029-2035.e5. doi:10.1016/j.celrep.2019.04.074

13. Uezumi A, Ikemoto-Uezumi M, Zhou H, et al. Mesenchymal Bmp3b expression maintains skeletal muscle integrity and decreases in age-related sarcopenia. *J Clin Investig*. 2020;131(1):e139617. doi:10.1172/jci139617

14. Urciuolo A, Quarta M, Morbidoni V, et al. Collagen VI regulates satellite cell self-renewal and muscle regeneration. *Nat Commun*. 2013;4(1):1964. doi:10.1038/ncomms2964

15. Uezumi A, Fukada S, Yamamoto N, et al. Identification and characterization of PDGFRα+ mesenchymal progenitors in human skeletal muscle. *Cell Death Dis*. 2014;5(4):e1186-e1186. doi:10.1038/cddis.2014.161

16. Lukjanenko L, Jung MJ, Hegde N, et al. Loss of fibronectin from the aged stem cell niche affects the regenerative capacity of skeletal muscle in mice. *Nat Med*. 2016;22(8):897-905. doi:10.1038/nm.4126

17. Mann CJ, Perdiguero E, Kharraz Y, et al. Aberrant repair and fibrosis development in skeletal muscle. *Skelet Muscle*. 2011;1(1):21-21. doi:10.1186/2044-5040-1-21

18. Chapman MA, Mukund K, Subramaniam S, Brenner D, Lieber RL. Three distinct cell populations express extracellular matrix proteins and increase in number during skeletal muscle fibrosis. *Am J Physiol-Cell Physiol*. 2017;312(2):C131-C143. doi:10.1152/ajpcell.00226.2016

19. Florio F, Vencato S, Papa FT, et al. Combinatorial activation of the WNT‐dependent fibrogenic program by distinct complement subunits in dystrophic muscle. *EMBO Mol Med*. 2023;15(12):EMMM202317405. doi:10.15252/emmm.202317405

20. Mueller AA, Velthoven CT van, Fukumoto KD, Cheung TH, Rando TA. Intronic polyadenylation of PDGFRα in resident stem cells attenuates muscle fibrosis. *Nature*. 2016;540(7632):276-279. doi:10.1038/nature20160

21. Ieronimakis N, Hays A, Prasad A, Janebodin K, Duffield JS, Reyes M. PDGFRα signalling promotes fibrogenic responses in collagen‐producing cells in Duchenne muscular dystrophy. *J Pathol*. 2016;240(4):410-424. doi:10.1002/path.4801

22. Micheli AJD, Laurilliard EJ, Heinke CL, et al. Single-Cell Analysis of the Muscle Stem Cell Hierarchy Identifies Heterotypic Communication Signals Involved in Skeletal Muscle Regeneration. *Cell Rep*. 2020;30(10):3583-3595.e5. doi:10.1016/j.celrep.2020.02.067

23. Lemos DR, Babaeijandaghi F, Low M, et al. Nilotinib reduces muscle fibrosis in chronic muscle injury by promoting TNF-mediated apoptosis of fibro/adipogenic progenitors. *Nat Med*. 2015;21(7):786-794. doi:10.1038/nm.3869

24. Lukjanenko L, Karaz S, Stuelsatz P, et al. Aging Disrupts Muscle Stem Cell Function by Impairing Matricellular WISP1 Secretion from Fibro-Adipogenic Progenitors. *Cell Stem Cell*. 2019;24(3):433-446.e7. doi:10.1016/j.stem.2018.12.014

25. Serrano AL, Baeza-Raja B, Perdiguero E, Jardí M, Muñoz-Cánoves P. Interleukin-6 Is an Essential Regulator of Satellite Cell-Mediated Skeletal Muscle Hypertrophy. *Cell Metab*. 2008;7(1):33-44. doi:10.1016/j.cmet.2007.11.011

26. Madaro L, Passafaro M, Sala D, et al. Denervation-activated STAT3–IL-6 signalling in fibro-adipogenic progenitors promotes myofibres atrophy and fibrosis. *Nat Cell Biol*. 2018;20(8):917-927. doi:10.1038/s41556-018-0151-y

27. Babaeijandaghi F, Kajabadi N, Long R, et al. DPPIV+ fibro-adipogenic progenitors form the niche of adult skeletal muscle self-renewing resident macrophages. *Nat Commun*. 2023;14(1):8273. doi:10.1038/s41467-023-43579-3

28. Zhang X, Ng YE, Chini LCS, et al. Senescent skeletal muscle fibroadipogenic progenitors recruit and promote M2 polarization of macrophages. *Aging Cell*. 2023;23(3):e14069. doi:10.1111/acel.14069

29. Lai Y, Ramírez-Pardo I, Isern J, et al. Multimodal cell atlas of the ageing human skeletal muscle. *Nature*. 2024;629(8010):154-164. doi:10.1038/s41586-024-07348-6

30. Cai C, Wan P, Wang H, et al. Transcriptional and open chromatin analysis of bovine skeletal muscle development by single‐cell sequencing. *Cell Prolif*. 2023;56(9):e13430. doi:10.1111/cpr.13430

31. Ma L, Meng Y, An Y, et al. Single‐cell RNA‐seq reveals novel interaction between muscle satellite cells and fibro‐adipogenic progenitors mediated with FGF7 signalling. *J Cachexia, Sarcopenia Muscle*. 2024;15(4):1388-1403. doi:10.1002/jcsm.13484

32. Thomas NT, Brightwell CR, Owen AM, et al. Satellite cells choreograph an immune cell-fibrogenic cell circuit during mechanical loading in geriatric skeletal muscle. *PNAS Nexus*. 2025;4(9):pgaf236. doi:10.1093/pnasnexus/pgaf236

33. Feng Y, Cui Z, Lu X, et al. Transcriptomics Dissection of Calorie Restriction and Exercise Training in Brown Adipose Tissue and Skeletal Muscle. *Nutrients*. 2023;15(4):1047. doi:10.3390/nu15041047

34. Saleh KK, Xi H, Switzler C, et al. Single cell sequencing maps skeletal muscle cellular diversity as disease severity increases in dystrophic mouse models. *iScience*. 2022;25(11):105415. doi:10.1016/j.isci.2022.105415

35. Stede TV der, Loock AV de, Turiel G, et al. Cellular deconstruction of the human skeletal muscle microenvironment identifies an exercise-induced histaminergic crosstalk. *Cell Metab*. 2025;37(4):842-856.e7. doi:10.1016/j.cmet.2024.12.011

36. Hao Y, Stuart T, Kowalski MH, et al. Dictionary learning for integrative, multimodal and scalable single-cell analysis. *Nat Biotechnol*. 2024;42(2):293-304. doi:10.1038/s41587-023-01767-y

37. Browaeys R, Saelens W, Saeys Y. NicheNet: modeling intercellular communication by linking ligands to target genes. *Nat methods*. 2019;17(2):159-162. doi:10.1038/s41592-019-0667-5

38. Jin S, Guerrero-Juarez CF, Zhang L, et al. Inference and analysis of cell-cell communication using CellChat. *Nat Commun*. 2021;12(1):1088. doi:10.1038/s41467-021-21246-9

39. Rodgers JT, King KY, Brett JO, et al. mTORC1 controls the adaptive transition of quiescent stem cells from G0 to GAlert. *Nature*. 2014;510(7505):393-396. doi:10.1038/nature13255

40. Cao J, Spielmann M, Qiu X, et al. The single cell transcriptional landscape of mammalian organogenesis. *Nature*. 2019;566(7745):496-502. doi:10.1038/s41586-019-0969-x

41. Lu H, Huang D, Ransohoff RM, Zhou L. Acute skeletal muscle injury: CCL2 expression by both monocytes and injured muscle is required for repair. *FASEB J*. 2011;25(10):3344-3355. doi:10.1096/fj.10-178939

42. Giordani L, He GJ, Negroni E, et al. High-Dimensional Single-Cell Cartography Reveals Novel Skeletal Muscle-Resident Cell Populations. *Mol Cell*. 2019;74(3):609-621.e6. doi:10.1016/j.molcel.2019.02.026

43. Geissmann F, Jung S, Littman DR. Blood Monocytes Consist of Two Principal Subsets with Distinct Migratory Properties. *Immunity*. 2003;19(1):71-82. doi:10.1016/s1074-7613(03)00174-2

44. Hume DA. The mononuclear phagocyte system. *Curr Opin Immunol*. 2006;18(1):49-53. doi:10.1016/j.coi.2005.11.008

45. Oprescu SN, Yue F, Qiu J, Brito LF, Kuang S. Temporal Dynamics and Heterogeneity of Cell Populations during Skeletal Muscle Regeneration. *iScience*. 2020;23(4):100993. doi:10.1016/j.isci.2020.100993

46. Chen YF. Temporal Single-Cell Sequencing Analysis Reveals That GPNMB-Expressing Macrophages Potentiate Muscle Regeneration. *Res Sq*. Published online 2024:rs.3.rs-4108866. doi:10.21203/rs.3.rs-4108866/v1

47. Saclier M, Yacoub‐Youssef H, Mackey AL, et al. Differentially Activated Macrophages Orchestrate Myogenic Precursor Cell Fate During Human Skeletal Muscle Regeneration. *Stem Cells*. 2013;31(2):384-396. doi:10.1002/stem.1288

48. Munder M, Eichmann K, Modolell M. Alternative Metabolic States in Murine Macrophages Reflected by the Nitric Oxide Synthase/Arginase Balance: Competitive Regulation by CD4+ T Cells Correlates with Th1/Th2 Phenotype. *J Immunol*. 1998;160(11):5347-5354. doi:10.4049/jimmunol.160.11.5347

49. Ruffell D, Mourkioti F, Gambardella A, et al. A CREB-C/EBPβ cascade induces M2 macrophage-specific gene expression and promotes muscle injury repair. *Proc Natl Acad Sci*. 2009;106(41):17475-17480. doi:10.1073/pnas.0908641106

50. Martinez-Pomares L, Reid DM, Brown GD, et al. Analysis of mannose receptor regulation by IL-4, IL-10, and proteolytic processing using novel monoclonal antibodies. *J Leucoc Biol*. 2003;73(5):604-613. doi:10.1189/jlb.0902450

51. Villalta SA, Nguyen HX, Deng B, Gotoh T, Tidball JG. Shifts in macrophage phenotypes and macrophage competition for arginine metabolism affect the severity of muscle pathology in muscular dystrophy. *Hum Mol Genet*. 2009;18(3):482-496. doi:10.1093/hmg/ddn376

52. Nawaz A, Bilal M, Fujisaka S, et al. Depletion of CD206+ M2-like macrophages induces fibro-adipogenic progenitors activation and muscle regeneration. *Nat Commun*. 2022;13(1):7058. doi:10.1038/s41467-022-34191-y

53. Liu L, Kim S, Buckley MT, et al. Exercise reprograms the inflammatory landscape of multiple stem cell compartments during mammalian aging. *Cell Stem Cell*. 2023;30(5):689-705.e4. doi:10.1016/j.stem.2023.03.016

54. Barai P, Chen J. Cytokine expression and cytokine‐mediated cell–cell communication during skeletal muscle regeneration revealed by integrative analysis of single‐cell RNA sequencing data. *J Cell Commun Signal*. 2024;18(4):e12055. doi:10.1002/ccs3.12055

55. Cutler AA, Pawlikowski B, Wheeler JR, et al. The regenerating skeletal muscle niche drives satellite cell return to quiescence. *iScience*. 2022;25(6):104444. doi:10.1016/j.isci.2022.104444

56. Keller‐Pinter A, Szabo K, Kocsis T, et al. Syndecan‐4 influences mammalian myoblast proliferation by modulating myostatin signalling and G1/S transition. *Febs Lett*. 2018;592(18):3139-3151. doi:10.1002/1873-3468.13227

57. Bentzinger CF, Wang YX, von Maltzahn J, Soleimani VD, Yin H, Rudnicki MA. Fibronectin Regulates Wnt7a Signaling and Satellite Cell Expansion. *Cell Stem Cell*. 2013;12(1):75-87. doi:10.1016/j.stem.2012.09.015

58. Cornelison DDW, Wilcox-Adelman SA, Goetinck PF, Rauvala H, Rapraeger AC, Olwin BB. Essential and separable roles for Syndecan-3 and Syndecan-4 in skeletal muscle development and regeneration. *Genes Dev*. 2004;18(18):2231-2236. doi:10.1101/gad.1214204

59. Mylona E, Jones KA, Mills ST, Pavlath GK. CD44 regulates myoblast migration and differentiation. *J Cell Physiol*. 2006;209(2):314-321. doi:10.1002/jcp.20724

60. Springer ML, Chen AS, Kraft PE, Bednarski M, Blau HM. VEGF Gene Delivery to Muscle. *Mol Cell*. 1998;2(5):549-558. doi:10.1016/s1097-2765(00)80154-9

61. Cheng N, Liu C, Li Y, et al. MicroRNA-223-3p promotes skeletal muscle regeneration by regulating inflammation in mice. *J Biol Chem*. 2020;295(30):10212-10223. doi:10.1074/jbc.ra119.012263

62. Summers C, Rankin SM, Condliffe AM, Singh N, Peters AM, Chilvers ER. Neutrophil kinetics in health and disease. *Trends Immunol*. 2010;31(8):318-324. doi:10.1016/j.it.2010.05.006

63. Grosicki GJ, Barrett BB, Englund DA, et al. Circulating Interleukin-6 is Associated with Skeletal Muscle Strength, Quality, and Functional Adaptation with Exercise Training in Mobility-Limited Older Adults. *J Frailty Aging*. 2020;9(1):57-63. doi:10.14283/jfa.2019.30

64. Pelosi L, Berardinelli MG, Forcina L, et al. Sustained Systemic Levels of IL-6 Impinge Early Muscle Growth and Induce Muscle Atrophy and Wasting in Adulthood. *Cells*. 2021;10(7):1816. doi:10.3390/cells10071816

65. Haddad F, Zaldivar F, Cooper DM, Adams GR. IL-6-induced skeletal muscle atrophy. *J Appl Physiol*. 2005;98(3):911-917. doi:10.1152/japplphysiol.01026.2004

66. Zhang J, Peng G, Chi H, et al. CD8 + T-cell marker genes reveal different immune subtypes of oral lichen planus by integrating single-cell RNA-seq and bulk RNA-sequencing. *BMC Oral Heal*. 2023;23(1):464. doi:10.1186/s12903-023-03138-0

67. Cortez KJ, Lyman CA, Kottilil S, et al. Functional Genomics of Innate Host Defense Molecules in Normal Human Monocytes in Response to Aspergillus fumigatus. *Infect Immun*. 2006;74(4):2353-2365. doi:10.1128/iai.74.4.2353-2365.2006

68. Luo X, Zheng H, Nai Z, et al. Identification of biomarkers associated with macrophage infiltration in non-obstructive azoospermia using single-cell transcriptomic and microarray data. *Ann Transl Med*. 2023;0(0):0-0. doi:10.21037/atm-22-5601

69. Ahmadzadeh K, Pereira M, Vanoppen M, et al. Multinucleation resets human macrophages for specialized functions at the expense of their identity. *EMBO Rep*. 2023;24(4):e57070. doi:10.15252/embr.202357070

70. Ochocka N, Segit P, Walentynowicz KA, et al. Single-cell RNA sequencing reveals functional heterogeneity of glioma-associated brain macrophages. *Nat Commun*. 2021;12(1):1151. doi:10.1038/s41467-021-21407-w

71. Kuswanto W, Burzyn D, Panduro M, et al. Poor Repair of Skeletal Muscle in Aging Mice Reflects a Defect in Local, Interleukin-33-Dependent Accumulation of Regulatory T Cells. *Immunity*. 2016;44(2):355-367. doi:10.1016/j.immuni.2016.01.009

72. Wickham H. ggplot2, Elegant Graphics for Data Analysis. *Use R!*. Published online 2016. doi:10.1007/978-3-319-24277-4

73. Xu S, Hu E, Cai Y, et al. Using clusterProfiler to characterize multiomics data. *Nat Protoc*. 2024;19(11):3292-3320. doi:10.1038/s41596-024-01020-z

74. Mootha VK, Lindgren CM, Eriksson KF, et al. PGC-1α-responsive genes involved in oxidative phosphorylation are coordinately downregulated in human diabetes. *Nat Genet*. 2003;34(3):267-273. doi:10.1038/ng1180

75. Subramanian A, Tamayo P, Mootha VK, et al. Gene set enrichment analysis: A knowledge-based approach for interpreting genome-wide expression profiles. *Proc Natl Acad Sci*. 2005;102(43):15545-15550. doi:10.1073/pnas.0506580102

76. Bankhead P, Loughrey MB, Fernández JA, et al. QuPath: Open source software for digital pathology image analysis. *Sci Rep*. 2017;7(1):16878. doi:10.1038/s41598-017-17204-5

77. Schmidt U, Weigert M, Broaddus C, Myers G. Cell Detection with Star-convex Polygons. *arXiv*. Published online 2018:265-273. doi:10.1007/978-3-030-00934-2_30
